# Children’s Glycemic Control: Mother’s Knowledge and Socioeconomic Status

**DOI:** 10.5539/gjhs.v5n6p214

**Published:** 2013-10-29

**Authors:** Abdulrahman Nasser Al-Odayani, Omar Zayyan Alsharqi, Ala’Eddin Mohammad Khalaf Ahmad, Abdulrahman Khazim Al-Asmari, Hussein Mohammad Al-Borie, Ameerah M.N. Qattan

**Affiliations:** 1 Department of Pediatrics, Prince Sultan Medical Military City, Riyadh, Kingdom of Saudi Arabia; 2 Faculty of Economics and Administration, King Abdulaziz University, Jeddah, Kingdom of Saudi Arabia; 3 Research Centre, Prince Sultan Medical Military City, Riyadh, Kingdom of Saudi Arabia

**Keywords:** children’s glycemic control, mother’s knowledge, socioeconomic status, Saudi Arabia

## Abstract

The present study was designed to examine the role of socioeconomic status (SES) of the mother’s knowledge about different aspects of diabetes and the glycemic control of type 1 children with diabetes. Samples were taken from successive admissions to the outpatient diabetes clinics in Prince Sultan Medical Military City (PSMMC), Riyadh, Saudi Arabia. A well designed questionnaire covering different aspects including demographic data, educational background, and socioeconomic status of the care providers was used to collect information from mothers of type 1 diabetes mellitus (T1DM) children. The questionnaire was designed on the basis of the Michigan diabetes knowledge scale and also on the basis of food habits of Saudi Arabia and it was validated. The questionnaire was completed after interviewing the mothers during visits to the PSMMC hospital. Every mother was asked with those particular questions. Glycemic control was assessed by glycosylated haemoglobin (HbA1c). The socio-demographic data of mothers was recorded by self-report. It was found that, there was significant variation in the knowledge of diabetes among mothers with different ages (p<0.05). Old age mothers and widowed mothers were better informed, however the difference was not statistically significant (p>0.05). No significant results were observed between family income and diabetes knowledge (p>0.05). However, a positive relationship was observed with higher income and higher knowledge. There was a significant association between mothers knowledge of diabetes and HbA1C level (r=-0.1739, p<0.05) indicating that, higher knowledge ultimately leads to greater control of HbA1c level. A significant association was also observed between education and HbA1c level (r=-0.2538, p<0.05) with children of mothers with higher level of education showing a better control of glycated haemoglobin levels. However, no significant association was found between monthly family income and HbA1C level. In conclusion, the current study illustrated that, mothers with more knowledge of diabetes and with better education were maintaining a better glycemic control of their children, irrespective of the socio-economic status. It was found that, to improve glycemic control and to decrease acute and chronic complications of diabetes in children, mother’s knowledge and education is needed.

## 1. Introduction

The incidence of type 1 diabetes (T1DM) has been increasing all over the world including Saudi Arabia. Recent reports indicate that some cities in Saudi Arabia have the highest reported incidence of childhood T1DM in the Middle East and North Africa region (Al-Herbish et al., 2008). Chronic complications of diabetes such as retinopathy, neuropathy, coronary heart disease and nephropathy, which are still present, might be prevented. This can be achieved by better awareness of the need for glycemic control.

Knowledge of disease and socioeconomic status (SES) of family/mother/caregiver plays an important role in the management of diabetes, especially type 1 diabetes in children. But there is paucity of data on these aspects in Saudi population (Raphael et al., 2003).

As a result of advancements in medical technology and treatment, the life span of children with chronic illness and physical disability has increased. However, chronic conditions may cause significant and permanent interference with the child’s physical, emotional, and social development and can affect family lifestyle and relationships. One of the major factors affecting the familial impact of chronic disease is the amount of time spent in the home managing the disease, and the amount of change in family life-style that must be made. Insulin-dependent diabetes mellitus (IDDM) is characterized by an almost immediate transfer of responsibility from health care officials to the family. The daily care and monitoring of diabetic control at home necessitates large expenditures of time and effort (Coclami et al., 1993).

Type I Diabetes is one of the most frequent chronic diseases that occur during childhood. Like other chronic diseases (asthma, cystic fibrosis, rheumatoid arthritis, epilepsy) children with diabetes and their families are focused to self-treatment and they become the directors of their own care.

Thus, the health care team should be the guides who set the stage, provides advice and oversight and helps to re-focus efforts when goals were not being met at all and canter around the patient and family (Brink et al., 2004). Rather than the diabetes health care teams being the only ones to initiate treatment, patients and their parents should be encouraged to interpret their blood glucose analysis results and try to adapt their daily activity and food habits accordingly (American Diabetes Association, 2006). On the other hand, poorly controlled diabetes may occur because mothers of children with type 1 diabetes probably fail to comprehend provided diabetes education. A recent study has shown that literacy and numerical skills of the mothers are related to better glycemic control in children with diabetes (Hassan & Heptulla, 2010).

Better knowledge of physiopathology of diabetes, availability of new insulin and devices, as well as education of health care providers are considered as important factors for improving self-care in children and adolescents with diabetes. These factors should be considered for the aim of improving metabolic control and quality of life of children and their family, and to reach to the ultimate aim of preventing macro-vascular and micro-vascular complications (Brown et al., 2004).

The primary data collection method was the research questionnaire, by asking the research respondents who represented by mothers, also from hospital main frame and personal interview. The secondary data were collected from different sources e.g. journals, articles, internet, and reports from the hospitals. Knowledge of diabetes was assessed using the modified Michigan Diabetes Research and Training Centre Diabetes Knowledge Test (MDRDT) (Fitzgerald et al., 1998), this modified MDRDT, consists of 29 questions. Eight questions to test general diabetes knowledge (e.g., “Which is the best method for testing blood glucose”), eight questions to test knowledge of insulin use (e.g., “If you are sick with the flu, which of the following changes should you make?”), five questions to test knowledge of complications, six question about knowledge of diabetic diet, one question about physical exercise and one question about glucagon use. The MDRDT scale was extended and 6 more questions were included for collection of information on the education, and specific food habits of Saudi Arabian population. Keywords used were Mother’s Knowledge, Socioeconomic Status, Children’s Glycemic Control, type 1 diabetes. Therefore the present study was undertaken to assess the relationship between knowledge, education and socioeconomic status of mothers and glycemic control in type 1 diabetic children.

## 2. Literature Review

In this section the researchers introduce the role of socioeconomic status in diabetes management, role of mothers in diabetes management, acute complication in children with type 1diabetes, chronic complications in children in diabetes, self management training of diabetic children, and diabetes management and education.

### 2.1 Role of Socioeconomic status in Diabetes Management

There has been interest in the relationship between socioeconomic status and health; individuals of higher socioeconomic status generally have better health and lower mortality rates than those who are less advantaged (Pickett et al., 2001). But what defines socioeconomic status? Socioeconomic status has been traditionally defined by the domains of education, income and occupation (Adler et al., 2002). Socioeconomic status can be measured at an individual or a group/aggregate level (for a community or neighbourhood) (Pickett et al., 2001); neighbourhood - level socioeconomic status is independently predictive of health outcomes after adjustment for individual status (Pickett et al., 2001).

Patients’ socioeconomic status was found to be positively correlated to the frequency of attendance in diabetic clinics, undergo routine blood tests (Brown et al., 2004) and self-monitor their blood glucose (BG) (Adams et al., 2003, Karter et al., 2000) less frequently than individuals of higher socioeconomic status. Furthermore, individuals with type 1 diabetes who are of low socioeconomic status have been found to be less accepting of preventative and health maintenance behaviour compared to those from higher social strata (Muhlhauser et al., 1998). As well, individuals with diabetes who are of low socioeconomic status may also develop hypoglycaemia in response to inadequate intake of food (Nelson et al., 1998), such factors may predispose these individuals to hypoglycaemia, and other severe life threatening events. The adjustment and ability to cope of mothers whose children are diabetics may be a key factor in reducing the impact on family life and in enhancing the child’s medical treatment. Additional measures may include personal wealth, material deprivation (such as lack of a vehicle or lack of home ownership) or perceived social status (National Institutes of Health, 2007).

### 2.2 Role of Mothers in Diabetes Management

The life-style and interpersonal relationships of the entire family are affected as the family must rearrange an established life-style to accommodate the requirements imposed by the diabetic regimen and treatment demands (Benolie et al., 1975). The situation is further complicated by the uncertainty of the final outcome of the illness. Several studies (Mellin et al., 2004; Pendley et al., 2002) have indicated that the mother is the person most involved with the coordination of care for the diabetic child. Parents’ education and their active involvement in their child’s diabetes self-management are crucial tools to achieve the desired goals. In paediatric type-1 diabetes mellitus (T1DM), the primary caregiver is mostly the mother (Delamater et al., 1990).

If the mother is the person most intimately bound to the details of the illness, any problems that she has in coping with the illness may affect the entire family. While studies analyzing the difficulties mothers have in managing a child with diabetes are few in number, the literature suggests that there are at least 11 aspects of care of the diabetic child that may be of concern to mothers: injections, diet, control, hypoglycaemic episodes, urine testing, finances, amount of help and support available, time demands, independence of the child, feeling that the illness is a stigma, and fears about the future (Banion et al., 1983).

Concern about hypoglycaemia, in particular, is higher with younger children and children who are younger at diagnosis. This is not surprising since mothers have more total responsibility for the care of younger children and it may be more difficult to assess or predict hypoglycaemic episodes in this age group. Knowledge about the signs, symptoms, and treatment of hypoglycaemia is important for all mothers but especially for those with young children. Blood glucose monitoring may be a helpful resource for mothers in differentiating confusing symptoms in young children (Grey et al., 2009).

### 2.3 Acute Complication in Children with Type 1 Diabetes

It was reported that, among the central complications of type I children’s diabetes is the Diabetic ketoacidosis (DKA) and severe hypoglycaemia. Although that both of them are life-threatening, yet they can be prevented (Bhatia et al., 1990). However, less is known about the incidence and predictors for DKA and severe hypoglycemia in the US population in the era after the Diabetes Control and Complications Trial (DCCT) (Levineet et al., 2001; Allen et al., 2001). Few of the previous studies examined predictors for DKA and severe hypoglycemia in children (Levine et al., 2001; Allen et al., 2001; Rosilio et al., 1998) and even fewer used a prospective design (Levineet et al., 2001; Allenet et al., 2001). US children with type 1 diabetes are at high risk for DKA (8 per 100 patient-years) and severe hypoglycemia (19 per 100 patient-years (Arleta et al., 2002).

Diabetic ketoacidosis often leads to an emergency department (ED) visit and hospital admission and contributes to the high costs of care for children with type 1 diabetes (Glaseret et al., 2001). Cerebral edema, a devastating complication of DKA, is one of the leading causes of mortality among children with type 1 diabetes (Robertset et al., 2001; Edge et al., 2001). Children with type 1 diabetes can be subjected to serious and acute complications among them: Poor glycemic control, family and school problems, low socioeconomic status, ethnicity, sex, and lack of adequate health insurance have been reported.

One of the most frequent diabetes-related complications as a result of insulin treatment is fall in blood glucose (Hypoglycemia). It is a serious complication that might be fatal especially in young children owing to the frequent glucose intake at this age. It occurs mostly at night. In severe hypoglcemia, the reported range of episodes is 4- 86 per year for both children and adolescent. The episode occurs most frequently at night. Symptoms of hypoglycaemia include shakiness, emotional instability, seizures, or unconsciousness. However, brain dysfunction as a sequel of repeated, prolonged hypoglycaemic episodes was reported as a minority (Schoenleet et al., 2002). However, Hypoglycemia can be prevented by guided erudition about the mentioned complications and methods of its prevention by careful food regimen and glucose intake observation.

### 2.4 Education and Counselling of Diabetic Children Mothers

As health care professionals are increasingly called upon to provide specialized services to children with diabetes and their families, it is important that emphasis be placed not only on the physical care needs of the child but also on psychosocial needs of the family. Efforts need to be directed toward lessening the possible disruption of family life. Facilitating the adjustment and ability to cope of mothers of children with diabetes may be a key factor in reducing the impact on family life and in enhancing the child’s medical treatment. Before health care providers can facilitate maternal coping with diabetes mellitus, they must first determine which aspects of the illness are the most difficult. As well as self-management requires that parents know how to prepare and give insulin injections, monitor blood glucose and urine kitones, record blood level values, manage diet including developing meal plans, manage exercise, and manage acute problems particularly hypoglycemia (Megeid et al., 2012).

## 3. Rationale for Research

To avoid the long term complications associated with diabetes and the immediate health risks associated with potentially life-threatening episodes of hypo- and hyperglycemia, mothers and children must be aware of issues related to glycemic control including diabetic diet, type of insulin in use, glucagon, diabetic complications and general knowledge of diabetes to maintain healthy blood glucose levels. The awareness may help in the proper control of diabetes in the population. The specific references only partially covered the children’s glycemic control: mother’s knowledge and socioeconomic status. Therefore, the present research attempts to fill a gap in the subject of children’s glycemic control in Saudi Arabia from the viewpoint of the mother’s. The research addressed some of the shortcomings in the literature, to investigate the mother’s knowledge and socioeconomic status.

## 4. Aim of the Research

The purpose of this study was to gather information from mothers of type1diabetic children in order to investigate the role of mother’s knowledge, education and SES of the Saudi family in relation to glycemic control of the children.

## 5. Research Methodology

### 5.1 Research Design

This is a descriptive, cross sectional study; the research participants were taken from successive admissions to the out patient’s diabetes clinics in PSMMC between 15 of August to 30 of September 2012. To be eligible for the study, participants were the primary caregiver of a child with diabetes. The selected child should age (a) between 6 months and 12 years of age, (b) diagnosed for at least a year with T1DM, (c) residing in the caregiver’s home, and (d) attending the clinic at least three times per year.

An informed consent was obtained from all participants who met our criteria and agreed to participate in the study. The participants were explained in detail about the study before obtaining their consent. The participants (all mothers in the current study) attended the same initial program at the same hospital, and were mothers of a child with diabetes.

All the children were hospitalized at the time of initial diagnosis. While in the hospital, the mothers received a standard in-hospital diabetes education provided by pediatric endocrinologists, health educators and the mothers were taught the role of a nurse. Included in this education were the following topics (i) an explanation of how the diagnosis has been made and the reasons for symptoms; (ii) a simple explanation of the uncertain cause of diabetes; (iii) the need for immediate insulin and how it works; (iv) what is glucose, normal blood glucose levels, and glucose targets; (v) practical skills (insulin injections, blood and urine tests, and reasons for monitoring); (vi) basic nutritional management; (vii) the effects of physical activity; (viii) a simple explanation of hypoglycemia and its treatment with glucose or sucrose; (ix) diabetes at home, at school, and during illnesses; and (x) emergency telephone contacts. In addition, (xi) each mother was given a booklet guideline “an educational Program for Children and Adolescents Suffering from Diabetes, their Parents and Teachers”.

### 5.2 The Research Population

The current research population were accurately specified in order to collect the required data for the research problem as explained earlier by Sekaran et al. (2000). The research population consists of mothers of children aged 6 months to 12 years who had type 1diabetes and who were receiving care at Prince Sultan Medical Military City (PSMMC) outpatient clinics.

### 5.3 Data Collection Methods

The data for this cross-sectional study were collected during regular office hours visits in the waiting area of outpatient’s diabetes clinics in PSMMC. And also from hospital main frame and personal interview. The secondary data were collected from different sources e.g. journals, articles, internet, and reports from the hospitals.

#### 5.3.1 Data Collection Instruments

The data for this cross-sectional study were collected during regular office hours in the waiting rooms of outpatient’s diabetes clinics in PSMMC, Department of Pediatrics. Knowledge of diabetes was assessed using the extended Michigan Diabetes Research and Training Center Diabetes Knowledge Test (MDRDT), (Fitzgerald et al., 1998), this extended MDRDT, consists of 29 questions. Eight questions to test general diabetes knowledge (e.g., “Which is the best method for testing blood glucose”), eight questions to test knowledge of insulin use (e.g., “If you are sick with the flu, which of the following changes should you make?”), five questions to test knowledge of complications, six question about knowledge of diabetic diet, one question about physical exercise and one question about glucagon use. The MDRDT scale was extended and 6 more questions were included for collection of information on the education, and specific food habits of Saudi Arabian population.

#### 5.3.2 Measure of Glycemic Control

Glycemic control was assessed by glycosylated hemoglobin (HbA1C) levels. Optimal glycemic control was defined as HbA1C values below 7.5; suboptimal glycemic control was defined as HbA1C values 7.5–9.0%, while poor control was defined as values greater than 9.0% (Rewers, 2007). The current study calculated the mean HbA1C value for each patient. All the HbA1C values taken every 3 months, and 1 year after diagnosis to the beginning of this study were used. Mother’s demographics information was obtained by self-report by using a questionnaire. They were asked to provide information about their age, level of education, monthly income, and marital status. To categorize the families’ SES, parents’ education level and current employment were recorded and analyzed according to the scale of salary as implemented in KSA. Participation was voluntary, and each mother signed an informed written statement of consent.

### 5.4 Research Questionnaire Design

Two types of questions are available for constructing the questionnaire, the open-ended and close-ended types (Vauset et al., 1993). Both types have their advantages and disadvantages. The questions addressed in this research were close-ended questions where the respondents are offered a set of answers and asked to select the answer that most closely represented their views. Questionnaire was designed on the basis of the Michigan diabetes knowledge scale and also on the basis of food habits of Saudi Arabia and it was validated.

### 5.5 Statistical Methods

Data were presented as median and ranges. The non-parametric Mann–Whitney and Kruskal–Wallis tests were used to compare groups as the variables did not have a normal distribution. Spearman rank correlation was used to examine relationships between the mother’s diabetes knowledge and HbA1C levels and between SES and HbA1C levels as there are two measurement variables and one hidden nominal variable. Significance were defined as a P<0.05. The statistical package SPSS was used for these analysis.

## 6. Results

In this section the researchers introduce the demographic data, general knowledge about diabetes, general knowledge of insulin and glucagon use, general knowledge of complications, general knowledge of diabetic diet and general knowledge of physical exercise.

### 6.1 Demographic Data

The detailed socio-demographic characteristics and responses to questions for each category are provided in Table 1. A total of 83 mothers fulfilled the inclusion criteria and voluntarily agreed to participate in this study. Only 2.4% of the participants were below 20 years of age and a majority (53.01%) was of middle age group (36-45 years). All the participants were married, and only one each was a divorcee (1.2%) and a widow (1.2%). All the participants reported their monthly family income. Of those 37 (44.57%) were having a monthly family income of <10,000 SR, and 25 (30.12%) were reported to have an income falling between 10,000 to 15,000 SR. The remaining 21 (25.30%) were having a monthly income >15000 SR (Table 2). Out of 83 mothers 6 (7.2%) were illiterate, while a majority 32 (38.55%) had attained college level education and 24 (28.91%) had completed secondary school level education (Table 3).

**Table 1:** Sociodemographic factors of mothers of Type 1 diabetic children

Variables	(N)	(%)	Correct Answers (Median; Range)
Age (In years)			
18- 20	2	2.4	15 (11-19)*
21-35	26	31.3	19.5 (10-25)
36-45	44	53.01	20 (9-26)
46-55	11	13.2	20 (15-25)
Marital status			
Married	81	97.59	20 (9-26)
Widowed	1	1.2	23
Divorced	1	1.2	18
Level of education			
Illiterate	6	7.2	17.5 (16-22)*
Elementary school	10	12.04	19.5 (12-23)
Secondary school	15	18.07	19 (10-23)
High school	32	38.55	20 (9-26)
College	20	24.09	21 (16-26)
Income			
<10,000	37	44.57	19 (10-25)
10,000-15,000	25	30.12	20 (10-26)
>15,000	21	25.30	20 (9-26)
Family Members			
From 1--5	18	21.68	19 (10-25)
From 6-10	59	71.08	20 (9-26)
More than 10	6	7.2	18 (17-23)
Occupation			
House wife	54	65.06	20 (10-26)
Govt. Employee	24	28.91	20 (9-25)
Business	0	0	0
Others	5	6.0	18 (15-24)
Residence Location			
Riyadh	69	83.13	20 (9-26)
Outside Riyadh	14	16.86	18.5 (10-26)
HbA1c			
<7.5 % (OGC)	2	2.4	22.5 (20-25)*
7.5-9 % (SGC)	26	31.3	20 (11-25)
>9 % (PGC)	55	66.26	20 (9-26)

* p<0.05 When compared to other groups; OGC-Optimal Glycemic Control; SGC-Suboptimal Glycemic Control; PGC-Poor Glycemic Control

**Table 2:** Monthly family income (SR) and level of glycated hemoglobin

Income	N	%	HbA1c level
<10,000	37	44.57	9.84 (± 1.56)
10,000-15,000	25	30.12	9.84 (±1.44)
>15,000	21	25.3	9.49 (±1.66)

**Table 3:** Literacy rate of mothers and their effect on HbA1c level (r = -0.1739, p<0.05)

Level of education	N	%	HbA1c level
Illiterate	6	7.2	10.32 (±2.06)
Elementary school	10	12.04	9.31 (±1.10)
Secondary school	15	18.07	10.31 (±1.27)
High school	32	38.55	9.74 (±1.73)
College	20	24.09	9.70 (±1.71)

### 6.2 General Knowledge about Diabetes

The awareness of mothers about each category related diabetes is given in Table 4. Less than 60% of the questions have been correctly answered by the mothers of diabetic. Eight out of 29 questions were regarding general knowledge of diabetes. 58.3 % of mothers (mothers) responded correctly for these questions, while 30% of the respondents failed to answer these questions correctly and the remaining were not sure of the answers.

**Table 4:** Overall responses of caregivers/mothers for the questions of each category during the survey

Categories	Number of Questions	Responses (Average)			Responses (%)		

		Correct	Wrong	Don’t know	Correct	Wrong	Don’t know
General knowledge of diabetes	**8**	48.375	25.125	9.375	58.28	30.27	11.30
General knowledge of Insulin use	**8**	73.375	8.5	1.125	88.40	10.24	1.36
General knowledge of Glucagon	**1**	70	5	8	84.34	6.02	9.64
General knowledge of Common Complications	**5**	63.92	12.88	6.167	77.01	15.51	7.43
General knowledge of Dietary	**6**	63.92	12.88	6.17	77.01	15.51	7.43
General knowledge of Physical Exercise	**1**	61	20	2	73.49	24.10	2.41

### 6.3 General Knowledge of Insulin and Glucagon Use

Majority of the respondents had a very good knowledge about the general use of insulin as more than 88% have correctly answered the queries on insulin. Whereas, an 11% mothers were not able to answer the questions on insulin correctly.

More than 84% of the mothers had a good knowledge of glucagon while 6% respondents had a wrong idea of the use of glucagon and 9.64% were not sure of the use of glucagon.

### 6.4 General Knowledge of Complications

Surprisingly around half of the mothers of diabetic children were unaware of the common complications of the diabetes. In this survey five questions were included for knowledge of complications, only 53.4% mothers responded correctly and more than 22% were found to be incorrect while 10.74% did not know anything about the complications.

### 6.5 General Knowledge of Diabetic Diet

Dietary knowledge is an important tool for management of diabetes and wellbeing of diabetic patients in long run. Most of the diabetic mothers were well aware of the importance of diabetic diet. Five questions were included in this category and more than 50% mothers answered these questions correctly while 22.15% were unaware about proper diabetic diet.

### 6.6 General Knowledge of Physical Exercise

Results demonstrated that most of the mothers (>73%) had a fairly good knowledge of the importance of physical activity in the management of diabetes, and a very small number of mothers (<3%) were unaware of the health benefits of physical exercise.

The knowledge of the mothers was examined using extended MDRTC scale. 29 points were available on this test, securing high scores indicating higher level of diabetic knowledge. In order to examine the effect of age factor on diabetic knowledge four groups were created and each group was compared. There was significant (p<0.05) variation in the knowledge of youngest group to the older groups. The older caregiver attained greater scores. No significant outcomes were observed between family income and diabetes knowledge (p>0.05) however a positive relationship was observed with higher income and higher knowledge. The data shows that high income woman is more conscious about diabetes as compared to low income woman. The median scores for mothers’ diabetes knowledge was significantly lower in illiterate mothers (p<0.05). However as the level of education increases the mothers tend to get higher scores. There was no significant difference in mothers diabetic knowledge in relation to the number of family members, occupation and residence location. Though statistically insignificant women having a moderate number (6-10) of family members, and those living in the capital Riyadh were found to be more concerned about diabetes than with either less (1-5) or more (>10) family members and then those living outside Riyadh.

**Figure 1:**
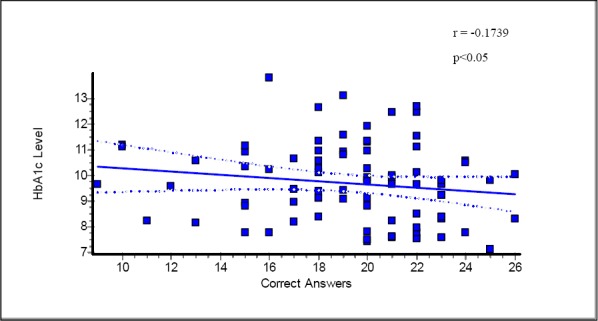
Regression plot showing relation between mother diabetic knowledge and blood HbA1c level

There was a significant association between mothers knowledge of diabetes and HbA1c level (r=-0.1739, p<0.05) indicating higher knowledge ultimately leads to greater control of HbA1C levels (Figure 1). A significant association also existed between level of education and HbA1C levels (Table 3) (r=-0.2538, p<0.05). Higher level of education leads to better glycemic control. On the other hand no significant association was found between monthly family income and HbA1C level (Table 2).

## 7. Research Discussion

The present study was designed to investigate the role of mother’s knowledge and SES of the family on glycemic control in diabetic children. As expected, children of mothers with higher knowledge scores tended to have lower HbA_1c_ levels. This finding is consistent with previous researches (Butler et al., 2008; Stalwood et al., 2006; Chaturvedi, 1996; Hassan, 2006) and supports the efficacy of diabetes education in helping mothers who are caring for an affected child to achieve better glycemic control. Of some concern was the fact that only 2.4% of the children had HbA_1c_ levels within the optimal glycemic control range, 31.32% were in suboptimal glycemic control range and 66.26% were in poor glycemic control range identified by this pediatrics clinic

Knowledge of diabetes was assessed using extended MDRTC scale. Most of the mothers were aware about the general diabetic knowledge, more than half (58.28%) of the mothers answered correctly, while the rest did not know or answered wrongly. Most of the mothers (>77%) were aware about how to check blood glucose. Almost every mother (88.40%) was aware about the use of insulin. However, few did not know more about insulin; may be due to lack of education and social barriers. Every mother had known well where she has to keep the insulin. 100% correct answers were recorded for this question. About glucagon, the knowledge of glucagon in mothers of type 1 diabetic children was comparable to insulin as majority of the mothers (84.34%) answered correctly. Poor awareness about diabetes complications was recorded among the mothers. Most of them were not aware about the chronic conditions of uncontrolled HbA1c level. In this survey poor results were obtained as only 53.41% responded correctly about the diabetic diet related questions although quite good correct responses (73.49%) were recorded about the effect of controlled physical exercise on blood glucose level.

Our study demonstrated that, there is an inverse relation between mothers’ diabetic knowledge and their HbA1c level. The caregiver having higher education, score high in knowledge test and that ultimately leads to better glycemic control (Rothman et al., 2004; Schillinger et al., 2005). Our study also demonstrates a positive but statistically insignificant relation between socioeconomic status and diabetes knowledge. This finding is in accordance with the findings of Sivagnanam et al. (1999) who found that, patients with diabetes have poor insight into their disease regardless of socioeconomic and educational status (Sivagnanam et al., 1999).

Those with greater income tended to have higher knowledge scores, indicating that families from lower socioeconomic situations may be at risk for knowledge deficits. Based on this information, interventions such as knowledge assessments and appropriate education may help to reduce any disparities in knowledge based on income, although not significant in our study, may increase glycemic control in all children.

Our study illustrated that mothers with more knowledge have children with better glycemic control, and low socioeconomic status is not significantly associated with higher levels of HbA1c. Improvement of mothers’ knowledge and family SES may improve glycemic control and ultimately decrease acute and chronic complications of diabetes in children. The mother plays the role of nurse and dietician and conveys the importance of diabetes treatment to her child (Brink et al., 2004) and communicates with members of the healthcare team during outpatient visits or through any communication media. Again, mothers are considered to be primary caregivers of children with IDDM (Kovacs et al., 1985). Therefore, to minimize the risk factors and medical urgencies it is very important for the members of the healthcare service to assess, correct, and/or expand the mother’s knowledge (Speight et al., 2001). Additionally, improving glycemic control through patient education may decrease the economic impact of diabetes (Panja et al., 2005) and we should educate the family members of the type 1 diabetes patient to get the best glycemic control.

## 8. Conclusion

Our study illustrated that mothers with more knowledge of diabetes and with better education were able to maintain a better glycemic control of their children, irrespective of the socio-economic status. Improvement of mothers’ knowledge and education may improve glycemic control and ultimately decrease acute and chronic complications of diabetes in children. Therefore, it is strongly suggested that, members of health care services assess, correct, and/or expand the mother’s knowledge of diabetes to facilitate the prevention of long term biomedical consequences. In addition, improving glycemic control through patient and care providers’ education may help in the decrease of economic impact of diabetes on the family and health care provider which is mainly supported by the Government of KSA. Furthermore it is also essential that a method of assessing the socioeconomic changes such as loss of income divorce and disability are incorporated into the routine questions that are put to the diabetic patient and the care provider. Counselling should be repeated any time that escalation of medicine is being discussed to reinforce the role of medication and to revisit patient diabetes knowledge.

### 8.1 Recommendation &Avenues for Future Research

The current levels of diabetes knowledge obtained in Saudi mothers in were only slightly better and indicating a need for further educational assessments and supplemental diabetes education as appropriate. Finally, this study supports similar findings linking age, knowledge and education levels with HbA1c profile, providing some insight into those most at-risk for less than optimum glycemic control.

In conducting this research an area was identified for further research and future study. This area includes participants among all Armed Forces Hospitals in order to get more samples and more generalized findings.

### 8.2 Research Limitations

1-Among other potentially unknown factors, low performance of the extended MDRTC may have been associated with the small sample size and short duration of study might be considered as a weakness.

2-One difficulty in conducting this research was that, very few studies in our country have been previously performed regarding mothers knowledge about diabetes care and its relation to socioeconomic and glycemic control. Although this will add to the originality and value of this study, the research didn’t added benefit of learning from others’ studies.
